# Editorial

**Published:** 2013-01-15

**Authors:** Shibal Bhartiya, Tarek Shaarawy, Tanuj Dada

‘We are what we repeatedly do. Excellence, therefore, is not an act but a habit.’

—Aristotle

Dear Friends,

This issue of the Journal of Current Glaucoma Pratice comes into your hans as we usher in a brand new year. Every new year, as is true for all new beginnings, brings with it the prospect of great joy and possobilities. We hope that it will, for all of us, be the much needed impects for both, personal and professional growth.

Jones et al report their experience with the preimplantation flow testing of Ahmed Glaucoma Valve (AGV) and the early postoperative clinical outcome and describe a simple in vivo gravity driven test to assess valve function after priming that may reduce hypotony rates. Perez et al report a case of nonpenetrating deep sclerectomy with iris incarceration post-Nd:YAG goniopuncture in which they documented spontaneous returning of the prolapsed iris during gonioscopy 3 hours after the onset of symptoms. They further report that, with an Argon laser iridoplasty, the iris returned completely to its position, and a wide Nd:YAG laser iridotomy prevented recurrences and good long-term intraocular pressure (IOP) control.

The prevalence, possible mechanisms and correlations IOP rise after anti-VEGF treatment are reviewed by Kampougeris et al. This review becomes extremely relevant since short-term IOP spikes are a fairly common and well recognized complication of anti-VEGF injections, and there is increasing interest among clinicians about its effects on the IOP in the long-term also. Duke et al evaluated the presenting visual acuity and ocular comorbidity in patients with primary open angle glaucoma in a Private Tertiary Eye Center in Nigeria. The original article provides a very useful insight into the management of the potentially blinding disease, bringing to light the unique challenges faced by the glaucoma practitioners in the region.

Stewart et al present the results of a prospective, multicenter, observational survey to evaluate the comfort upon instillation of timolol hemihydrate compared to timolol maleate with potassium sorbate. The survey demonstrates that the former is associated with less stinging/burning and tearing, a finding which has the potential to greatly influence patient compliance.

Molecular diagnostics and genetic counseling in primary congenital glaucoma is addressed by Faiq et al in a comprehensive review of the genes involved in etiology and pathogenesis of PCG. The authors also discuss the scope of many molecular biology techniques in the investigation of the molecular mechanisms and enhanced understanding of the disease, which may prove to be of great benefit in the clinical setting for patient management, risk assessment and genetic counseling.

Ansari assesses whether the IOP in selective laser trabeculoplasty (SLT)-treated eyes is maintained following subsequent phacoemulsification and lens implant (phaco + IOL) in a retrospective single center review thereby reporting that IOP reduction with SLT is not significantly affected by subsequent phaco + IOL in patients with OAG.

We hope, this issue of the JOCGP provides you information that is relevant and useful in your clinical practice.

As always, we look forward to your feedback, criticisms and suggestions.

Best wishes

**Shibal Bhartiya**

**Tarek Shaarawy**

**Tanuj Dada**

